# 5-Formyltetrahydrofolate in a Cohort of Pregnant Women Following Folic Acid Supplementation

**DOI:** 10.1021/acsomega.5c00251

**Published:** 2025-04-28

**Authors:** Miruna Sudharshani Kalaimani Rabindrakumar, Veranja Karunaratne, Carukshi Arambepola, Vijay Pal Singh, Sharmila Jayasena, V. Pujitha Wickramasinghe, Tharanga Thoradeniya

**Affiliations:** † Department of Biochemistry and Molecular Biology, Faculty of Medicine, University of Colombo, 25 Kynsey Rd, Colombo 00800, Sri Lanka; ‡ Department of Chemistry, Faculty of Science, University of Peradeniya, University of Old Galaha Rd, Peradeniya 20400, Sri Lanka; § Department of Community Medicine, Faculty of Medicine, University of Colombo, 25 Kynsey Rd, Colombo 00800, Sri Lanka; ∥ CSIR-Institute of Genomics & Integrative Biology, Academy of Scientific and Innovative Research, New Delhi 110025, India; ⊥ Department of Paediatrics, Faculty of Medicine, University of Colombo, 25 Kynsey Rd, Colombo 00800, Sri Lanka

## Abstract

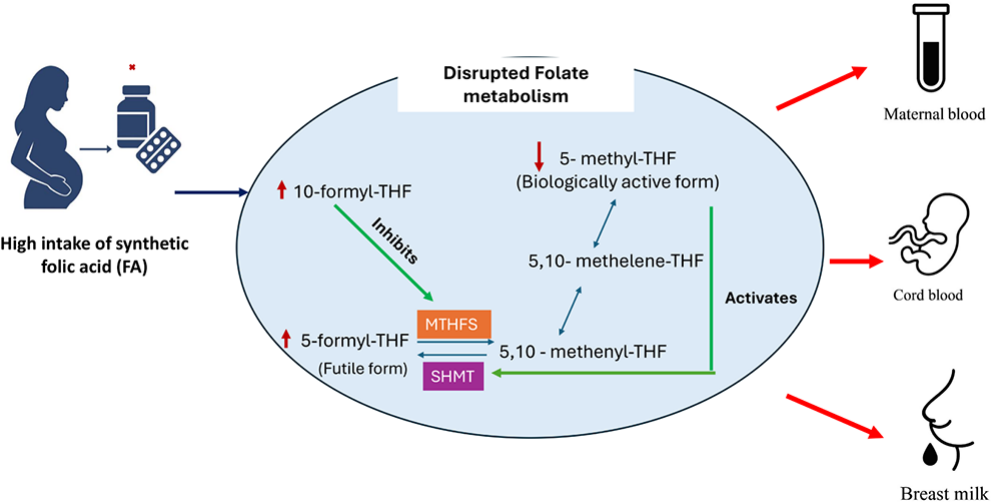

Disrupted folate metabolism related to high synthetic folic acid (FA) intake is a matter of contention. FA and its metabolites play a critical role in DNA synthesis and methylation, and inadequate or imbalanced folate status is strongly associated with neural tube defects and other adverse health outcomes. We determined different folate forms using the LC/MS-MS method in maternal blood, cord blood, and breast milk of women (*n* = 50) following the National Iron-Folic Acid (FA) Supplementation Program. High concentrations of 5-formyltetrahydrofolate (5-formyl-THF) (range: 40.9–222.7 nmol/L) were observed throughout pregnancy, in cord serum, and breast milk. Levels of 5-formyl-THF rapidly increased (mean difference: 181.6 nmol/L) after 4–6 weeks of supplementation with 1 mg of FA/day and subsequently decreased (mean difference: 139.8 nmol/L) upon continuous supplementation at 400 μg of FA/day. The rapid increase in 5-formyl-THF following supplementation was higher (*p* < 0.001) in women with methylenetetrahydrofolate reductase (MTHFR) C677T polymorphism. 5-methyl-THF (range: 11.4–56.8 nmol/L) and FA (range: 26.8–39.6 nmol/L) were detected only in breast milk. MTHFR 677CT/TT genotypes were associated with lower 5-methyl-THF (*p* < 0.001) and higher (*p* < 0.001) 5-formyl-THF in breast milk. At baseline, 48% had low (<340 nmol/L) RBC folate, but the concentrations continuously increased (*p* < 0.001) across pregnancy despite the different FA doses. The unusual observation of high 5-formyl-THF, the futile folate form, and its modulation with FA dose and genetic polymorphism merit further investigation to elucidate the population dynamics and possible physiological/clinical significance while questioning the utility of RBC folate as a biomarker of usable folate forms.

## Introduction

Despite decades of public health interventions, folate deficiency and insufficiency remain public health concerns in developing countries as well as in the developed world. Impaired folate metabolism during pregnancy is directly linked to neural tube defects, megaloblastic anemia, preterm birth, low birth weight, and neonatal folate deficiency. It is also associated with cardiovascular disease, dementia, and various cancers.^1−3^ Folic acid (FA), the fully oxidized, stable synthetic form of folate, is widely used as a supplement or in food fortification to overcome these problems. Due to its anticipated clinical benefits, perceived safety, and low cost, it is widely used globally across all population groups, including pregnant women.^4^

The use of FA has been controversial, although the benefit of its use in reducing the incidence of neural tube defects is unquestionable.^5^ Concerns have been raised about a high intake of synthetic FA, such as the increased risk of disrupted metabolism, anemia, cognitive impairment, and, more importantly, the appearance of unmetabolized FA (UMFA) due to the limited capacity of the human gut to reduce FA.^6,7^ Evidence on the mechanisms of these adverse effects of FA supplementation is limited. Whether the effects are solely due to FA exposure alone or an increase in any one or more of the biological forms of folate is not yet clear.

**Figure 1:**
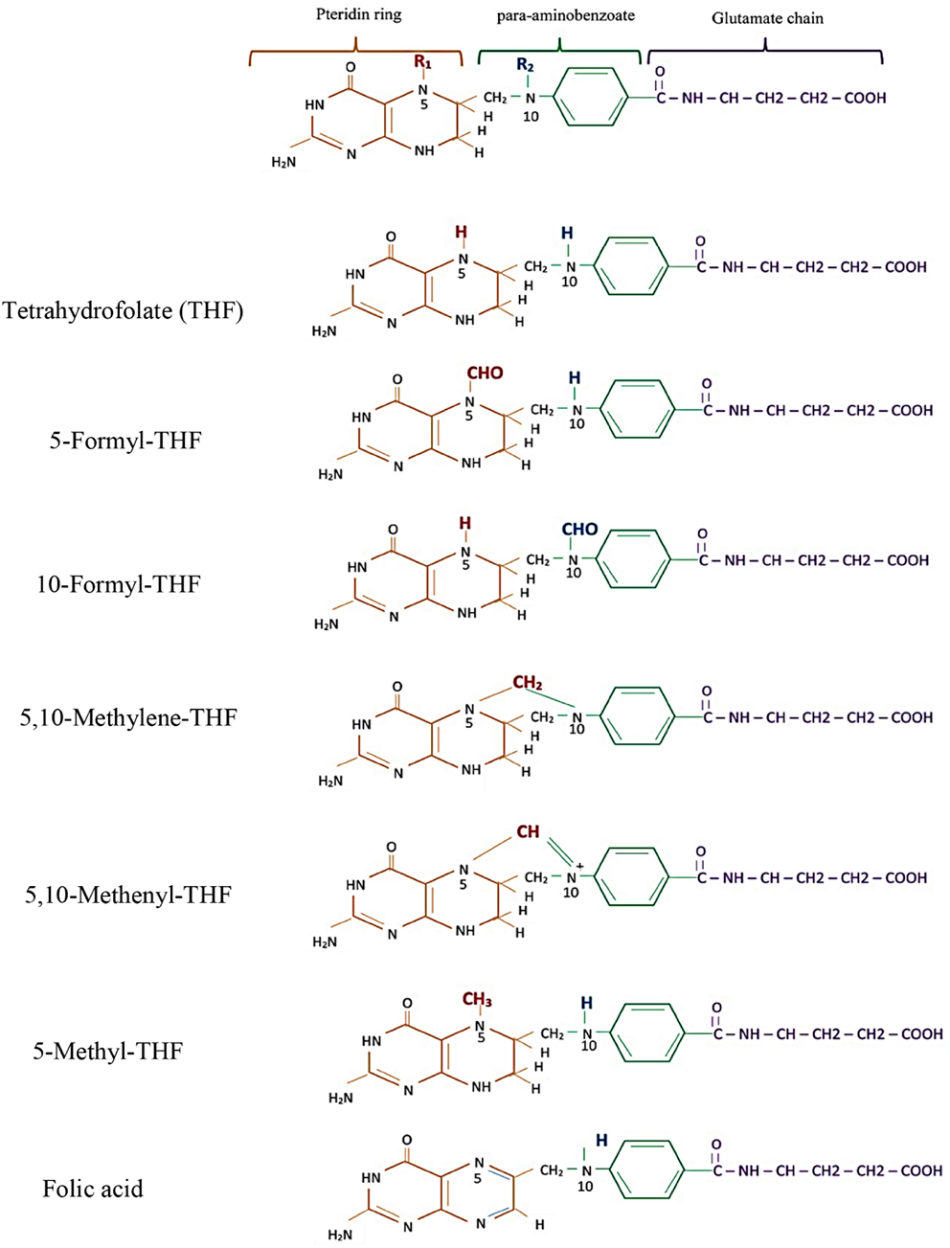
Chemical structures of folates. The folate forms differ in the oxidation state of the pteridine ring and in the one-carbon substitution at the fifth and/or 10th positions of nitrogen atoms.

Folate is a group of B vitamins consisting of tetrahydrofolate (THF), 10-formyl-THF, 5,10-methylene-THF, 5-formyl-THF, and 5-methyl-THF, which are the different biological forms of folate (Figure 1) involved in folate metabolism. These forms have different functions and have gained much interest at present.^8^ An analysis of postfortification data from the 2011–2016 US NHANES suggests that demographic, physiological, and lifestyle characteristics influence the concentrations of different folate forms.^9^ Further, variations in the distribution of folate forms were observed when there were mutations in key folate-metabolizing enzymes.^10^

However, all of this information is from populations susceptible to having high folate levels and where mandatory or voluntary fortification takes place. To the best of our knowledge, no studies have been conducted in a population where folate deficiency is a public health problem and where population dynamics are distinct from Western populations, particularly in Asia.^11^ Further, Sri Lanka provides 1 mg/day of FA, a dose higher than that recommended by the World Health Organization (WHO) (400 μg/day), via the National Supplementation Programs for Women of Childbearing Age and Pregnant Women.^12,13^ Although routine analysis indicates that total folate levels increase after FA supplementation, it does not differentiate between UMFA or any other folate forms.^14^ With this background, we aimed to describe the folate forms in maternal blood, cord blood, and breast milk, identify the presence of circulatory UMFA in a cohort of pregnant women following the National FA Supplementation Program in Sri Lanka, and determine the association of these folate metabolites with genetic variants of key folate-metabolizing enzymes.

## Study Design and Methods

### Study Design and Participants

The study samples were drawn randomly from a clinic-based follow-up study conducted among Sri Lankan pregnant women to assess anemia, iron, and folate status following FA and iron supplementation. The detailed study design was reported in our earlier work.^15^ Healthy pregnant women aged 18–36 years, attending antenatal clinics in the Colombo Municipal Council area for their booking visit during September 2015–June 2016, were invited for participation. The subsample drawn for the present analysis was *n* = 50 and complied with the minimum number of participants required for assessing the difference in low folate stores before (51%) and after (21%) supplementation (based on the initial findings of the same cohort), with an α error of 0.05 and 80% power. The study was carried out in accordance with the guidelines laid down in the Declaration of Helsinki, and the study protocol (EC-15-006) was approved by the Ethics Review Committee of the Faculty of Medicine, University of Colombo, Sri Lanka. Informed written consent was obtained from all of the participants before starting the study procedures.

Folate forms were measured in nonfasting blood samples at baseline, at gestational age (ga) < 12 weeks, before starting any supplementation; in early pregnancy (ga 12–17 weeks) following 4–6 weeks of 1 mg of FA/day; in late pregnancy (ga > 34 weeks) following 16–20 weeks of iron-FA (400 μg/day); at delivery, in cord blood, and in 6–8-week postpartum breast milk samples. Nearly 98% of the women complied with supplementation, and all took FA tablets after meals between 2000 and 2100 h.

### Laboratory Analysis

#### Sample Preparation

Blood samples were collected into gel separation tubes and ethylenediaminetetraacetic acid (EDTA) tubes shielded from light and processed within 3 h of collection. Aliquots of serum and whole blood were transferred into cryovials wrapped in aluminum foil under yellow light and stored at −80 °C for batch analysis of folate forms and RBC folate analysis. The remaining blood was used for DNA extraction using the Wizard Genomic DNA Purification Kit (Cat No. A1120, A1125, Promega Corporation, USA). Breast milk samples were collected in the middle of a feed, at least 2 h after the previous breastfeeding. The samples were collected by hand expression under yellow light into sterile polypropylene centrifuge tubes wrapped in aluminum foil and stored at −80 °C for batch analysis of folate forms.

#### RBC Folate Assay

RBC folate was assessed in maternal and cord blood using chemiluminescent microparticle immunoassay (Abbott *i*1000). The inter- and intra-assay coefficients of variation (CV) were <5% and <3%, respectively. Experimental standards were maintained by daily calibration with standard quality control (QC) (Lyphochek Immunoassay Plus Control, Bio-Rad, USA) and pooled serum analysis.

#### Folate Form Analysis via Liquid Chromatography–Mass Spectrometry (LC-MS/MS)

**Figure 2:**
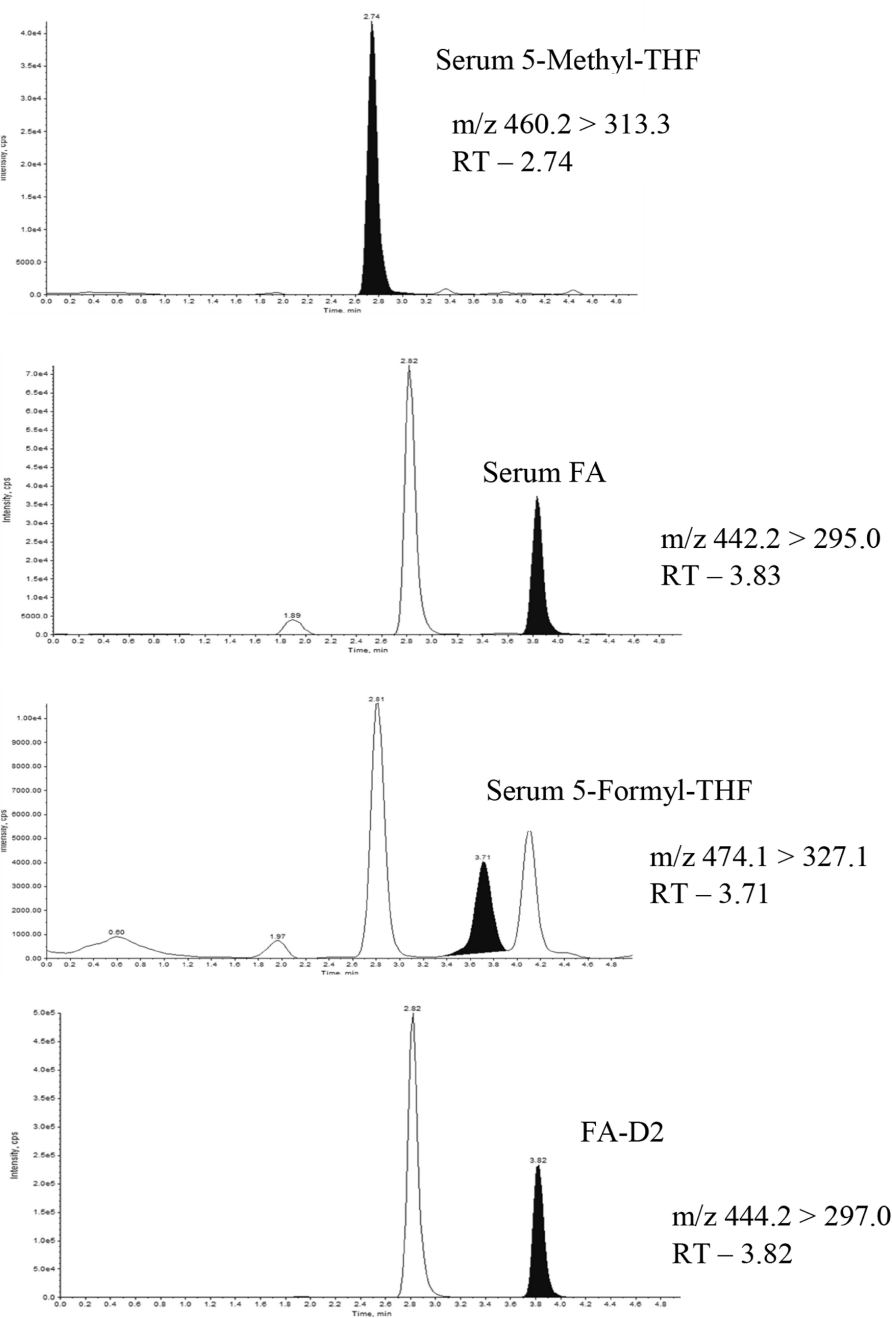
Representative LC-MS/MS chromatograms in serum.

**Figure 3:**
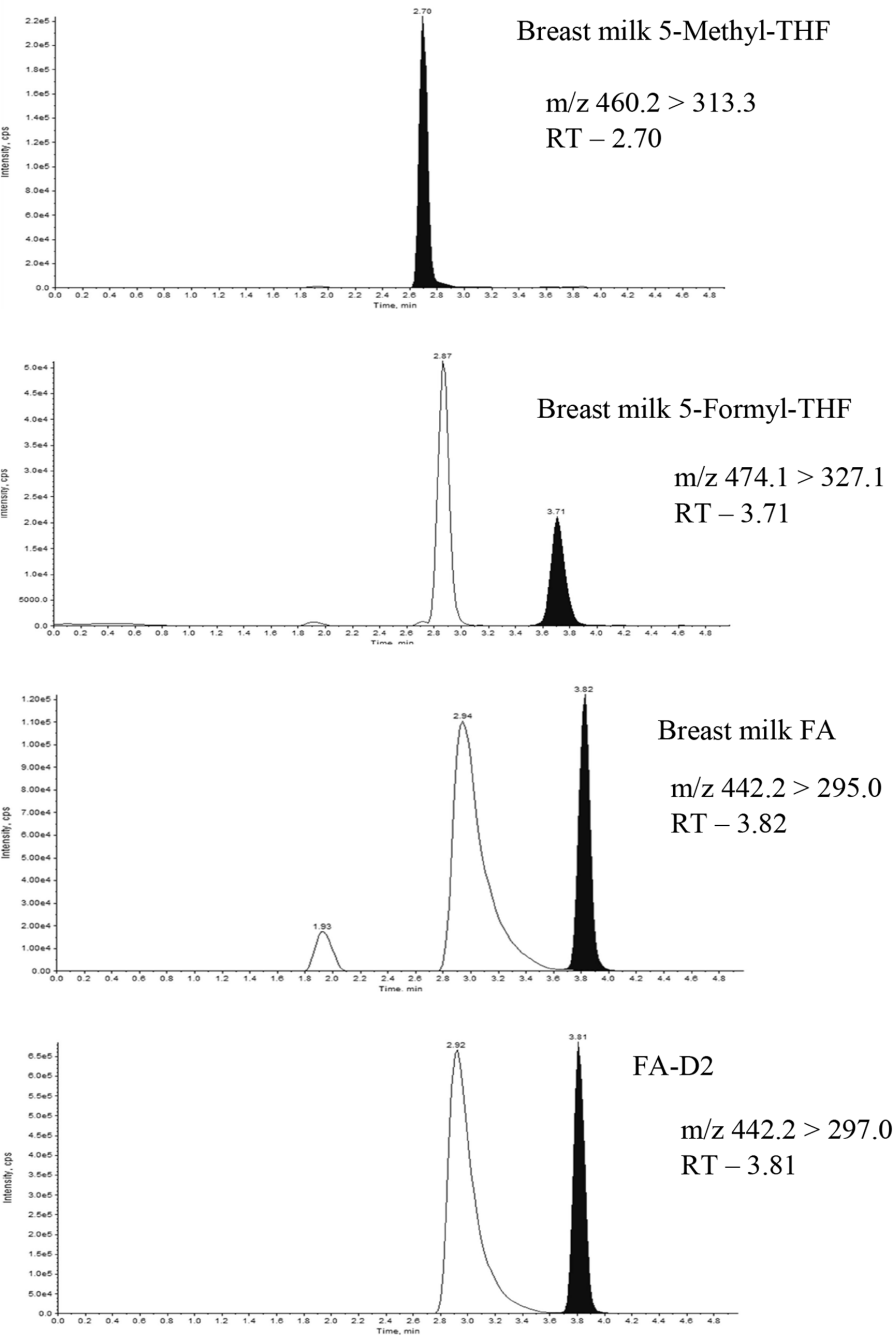
Representative LC-MS/MS chromatograms in breast milk.

The concentrations of 5-methyl-THF, 5-formyl-THF, and FA were measured based on the method developed by Doyle and Riley in 2017, using state-of-the-art LC-MS/MS at the Dabur Research Foundation, Ghaziabad, Uttar Pradesh, India.^16,17^ The detailed analytical protocol is given in the Supporting Information. In brief, folates were extracted from serum and breast milk with 10% orthophosphoric acid in acetonitrile. The proteins were precipitated, and the supernatant was transferred to a clean tube. 100 μL of ammonium acetate buffer containing 1 mg/mL of mercaptoethanol was added to the supernatant. Mercaptoethanol, a strong reducing agent, maintains a reducing environment to prevent the oxidative degradation of folates. It stabilizes labile folate forms, such as 5-methyltetrahydrofolate, for accurate LC-MS/MS quantification. Additionally, it denatures proteins by breaking disulfide bonds, preventing interference and folate rebinding. The sample was vortexed and transferred into autosampler vials for injection into the LC-MS/MS system (SCIEX QTRAP–5500). Quantification was achieved with the use of internal standards and a calibration curve. The concentrations of 5-methyl-THF, 5-formyl-THF, and FA were analyzed in positive electrospray mode (Table 1). The multiple reaction monitoring (MRM) chromatograms of all analytes in serum and breast milk are shown in Figures 2 and 3. An MRM chromatogram is a tandem mass spectrometry (MS/MS) plot showing the intensity of specific fragment ions from specific parent ions (specific folates) over time, enabling sensitive and selective quantification of target analytes in complex samples, including serum. The limits of detection (LODs) and limits of quantification (LOQs) for all folate forms were 0.6 and 1.4 nmol/L, respectively. The method was validated by assessing the linearity, precision, accuracy, and recovery of the analytical method. The coefficient of linear regression in serum and breast milk for all three folate forms was *r*^2^ > 0.992. The detailed percentages of precision obtained for each analyte in serum and breast milk can be found in Tables S1 and S2, respectively. The mean recoveries for 5-methyl-THF, 5-formyl-THF, and FA were >80%.

**Table 1:** Optimized MS/MS Parameters for the Determination of Folate Forms^a^

Analyte	MRM transition m/z	DP (V)	CE (V)
5-methyl-THF	460.2 → 313.3	19	19
5-formyl-THF	474.1 → 327.1	27	27
folic acid	442.2 → 295.0	17	17
folic acid D2	444.2 → 297.0	35	35

Source parameter	Optimized value
source temperature (°C)	650
ionization voltage (V)	5500
ion source (GS1)	50
ion source (GS2)	55
curtain gas (CUR)	25

a MRM transition—selected pair of a precursor ion (parent ion) and its corresponding fragment ion; DP, declustering potential (the voltage applied at the interface between the ion source and the mass spectrometer. It helps remove solvent molecules or weakly bound clusters from the ions, allowing cleaner, individual ions to enter the mass analyzer for improved signal quality); CE, collision energy (the amount of energy applied to fragment the precursor ion in the collision cell).

#### DNA Isolation and Genotyping

The extracted DNA was >20 kb in all instances, and thus free of RNA contamination. The methylenetetrahydrofolate reductase (MTHFR) C677 > T polymorphism was genotyped using the polymerase chain reaction with restriction fragment length polymorphism (PCR/RFLP) method.^18^ The dihydrofolate reductase (DHFR) 19-bp deletion polymorphism was assessed using high-resolution melt (HRM) curve analysis.^19^

### Statistical Analysis

Statistical tests were performed using SPSS (Version 21.0). The one-sample Kolmogorov–Smirnov test was used to check the normality of the outcome variables. Accordingly, the concentrations of RBC folate, 5-formyl-THF in serum and breast milk, and 5-methyl-THF in breast milk were all normally distributed and thus presented as means with standard deviation (SD). FA in breast milk was detected only in six samples, was log-transformed, and presented as a geometric mean with geometric standard deviation. The concentrations of folate forms in serum and breast milk were further compared with MTHFR C677T and DHFR 19-bp deletion genotypes of the women using the Student’s *t*-test. According to WHO guidelines for pregnant women, folate deficiency was defined as RBC folate of <342.2 nmol/L.^20^

## Results

### Baseline Folate Forms Based on the Women’s Folate Stores

Baseline characteristics of women are listed in Table 2. At baseline, 24/50 women had low folate stores (RBC folate < 342.2 nmol/L). Their maternal characteristics were not significantly different from those having sufficient folate stores at baseline. A high concentration of 5-formyl-THF was observed at baseline, while the concentrations of 5-methyl-THF and FA in serum were below the limit of detection (<0.6 nmol/L) at all visits. Comparatively, a lower 5-formyl-THF concentration was noted among women with low folate stores. Breast milk folate forms were not significantly associated with folate stores.

**Table 2:** General Characteristics, Genetic Variants, Dietary Folate Intake, and Serum Folate Forms at Baseline in Relation to the Folate Status of Women at Baseline

Factors	All *N* = 50	Women with low folate stores *N* = 24	Women with sufficient folate stores *N* = 26	*p*-value
age, years	25.2 ± 5.3	23.3 ± 4.0	27.0 ± 5.7	0.011
gestational age (week)	9 ± 1	9 ± 1	9 ± 1	0.185
BMI, kg/m^a^	23.1 ± 4.5	23.7 ± 4.3	22.6 ± 4.7	0.409
gravidity				
primigravida *n* (%)	16 (32.0)	10 (62.5)	06 (37.5)	0.159
multigravida *n* (%)	34 (68.0)	14 (41.2)	20 (58.8)	
MTHFR 677C → T polymorphism^a^	84:16	87.5:12.5	80.8:19.2	0.399
CC:CT:TT, %				
C:T	0.92:0.08	0.94:0.06	0.89:0.11	
DHFR 19-bp deletion polymorphism	58:38:4	62.5:29.2:8.3	53.8:46.2	0.194
WW:DW:DD, %				
W:D	0.77:0.23	0.77:0.23	0.77:0.23	
dietary intake				
folate-rich food serving/day	3.3 ± 2.1	3.1 ± 1.8	3.4 ± 2.4	0.480
folate forms at baseline, nmol/L				
RBC folate	372.8 ± 130.1	281.8 ± 37.7	457.5 ± 127.6	<0.001
serum 5-methyl-THF^b^	ND	ND	ND	
serum 5-formyl-THF	41.5 ± 20.4	37.6 ± 22.2	45.3 ± 18.4	0.187
serum folic acid^b^	ND	ND	ND	
breast milk folate forms, nmol/L				
5-methyl-THF^c^	27.9 ± 17.0	54.2 ± 28.1	65.3 ± 42.8	0.291
5-formyl-THF	59.8 ± 36.5	27.9 ± 17.9	28.1 ± 16.3	0.937
folic acid^d^	33.5 ± 4.8	2.8 ± 9.5	5.2 ± 12.5	0.449

a MTHFR and DHFR allele frequencies were in accordance with Hardy–Weinberg equilibrium (HWE) in control subjects (*p* > 0.05).

b Below the limit of detection (LOD) < 0.6 nmol/L.

c 5-Methyl-THF was below the LOD in 10 samples. Hence, the data are presented for 40 samples.

d Data are presented for six samples in which FA was detected. The Student’s *t*-test was used to compare continuous variable means between groups. The Chi square test was used to compare the percentages of the categorical variables between groups. ^*^ Significant difference *p* < 0.001. THF: tetrahydrofolate.

### 5-Formyl-THF Concentration Following FA Supplementation

A rapid increase (*p* < 0.0001) was noted in the concentration of serum 5-formyl-THF (mean difference: 179.9 nmol/L) following 4–6 weeks of 1 mg of FA supplements, despite their folate stores (Table 3). Subsequently, the concentration of serum 5-formyl-THF was reduced (*p* < 0.0001) following 16–20 weeks of iron-FA supplements containing 400 μg of FA. In contrast to 5-formyl-THF, the concentration of RBC folate significantly (*p* < 0.001) and continuously increased throughout pregnancy, despite the difference in the dose of FA at each visit. Of the 24 women with RBC folate < 342.2 nmol/L, 21 restored their folate stores, with RBC folate levels of above 340 nmol/L following supplementation.

**Table 3:** Concentration of Folate Forms in Maternal and Cord Serum and Breast Milk in Relation to Maternal MTHFR C677T and DHFR 19-Bp Deletion Genotypes^a^

	All	MTHFR C677T genotype		DHFR 19-bp deletion genotype	
nmol/L	(*N* = 50)	CC (*n* = 42)	CT/TT (*n* = 08)	WW (*n* = 29)	WD/DD (*n* = 21)
Maternal serum					
5-methyl-THF^b^	ND	ND	ND	ND	ND
5-formyl-THF					
Baseline	41.6 ± 20.5	40.1 ± 20.0	47.3 ± 24.1	45.5 ± 20.7	36.6 ± 19.6
4–6 weeks FA	220.7 ± 114.8^c^	208.9 ± 102.3	298.4 ± 135.5^d^	248.2 ± 101.4	182.5 ± 123.7
16–20 weeks	77.0 ± 40.9^e^,^f^	77.1 ± 40.9	102.1 ± 43	91.4 ± 45.9	66.6 ± 30.9
Iron-FA					
At delivery^g^	81.6 ± 40.7	77.5 ± 38.9	99.3 ± 47.5	93.6 ± 45.2	65.9 ± 28.4
FA^b^	ND	ND	ND	ND	ND
Cord serum^g^					
5-methyl-THF^b^	ND	ND	ND	ND	ND
5-formyl-THF	66.8 ± 25.2	69.6 ± 24.1	60.0 ± 31.4	66.1 ± 44.6	67.7 ± 27.5
FA^b^	ND	ND	ND	ND	ND
Breast milk					
5-methyl-THF^b^	34.3 ± 11.6	36.4 ± 10.7	21.4 ± 9.6	33.2 ± 11.8	36.4 ± 11.6
5-formyl-THF	60.0 ± 36.6	54.3 ± 35	89.5 ± 32.0	58.6 ± 34.1	62.0 ± 40.7
FA^b^	33.2 ± 2.7	33.2 ± 2.7	ND	33.6 ± 2.5	32.5 ± 3.0

a Data are expressed in mean ± SD. Abbreviation: 5-methyl-THF, 5-methyltetrahydrofolate; 5-formyl-THF, 5-formyltetrahydrofolate; FA, folic acid; ND, not detected; MTHFR, methylenetetrahydrofolate; DHFR, dihydrofolate reductase; W, wild type; D, deletion.

b Below the limit of detection (1.3 nmol/L) in maternal serum. In breast milk, 5-methyl-THF was detected in 41 samples. FA was detected in six samples. Geometric mean ± SD is presented for those with detectable concentrations of FA. Paired *t*-test was used to compare the means of folate forms between each visits.

c Significant difference (*p* < 0.001) between baseline and 4–6 weeks of 1 mg-FA/day.

d Significant difference higher (*p* < 0.001) in MTHFR CT than CC polymorphism.

e Significant difference (*p* < 0.001) between 4 and 6 weeks of 1 mg-FA/day and 16–20 weeks of iron-FA.

f Significant difference (*p* < 0.001) between baseline and 16–20 weeks of iron-FA.

g Data available for 39 paired maternal and cord samples at the time of delivery.

There was no significant (*p* > 0.05) association between the genotypes and the baseline folate forms. The stratified analysis shows that the rapid increase of 5-formyl-THF following 4–6 weeks of 1 mg of FA was higher (*p* = 0.044) in women carrying the T allele compared to women who were homozygous for the C allele for the MTHFR gene. Whereas, an opposing nonsignificant tendency (*p* = 0.098) was noted in women carrying 19-bp deletion alleles compared with women carrying the wild-type allele for the DHFR gene. However, no associations were noted with MTHFR C677T (*p* = 0.376) or the DHFR 19-bp deletion genotype (*p* = 0.312) and the subsequent reduction of serum 5-formyl-THF following 16–20 weeks of 400 μg of FA in iron-FA supplements. Further, no associations were observed between maternal genotypes and cord serum folate forms.

However, in breast milk, the concentration of 5-methyl-THF was higher (*p* = 0.011) in women who were homozygous for the C allele (36.4 ± 10.7 nmol/L) than in women carrying the T allele (21.4 ± 9.6 nmol/L) for the MTHFR gene. In contrast, the 5-formyl-THF concentration in breast milk was lower (*p* = 0.028) in women who were homozygous for the C allele (54.3 ± 35 nmol/L) than in women carrying the T allele (89.5 ± 32.0 nmol/L) for the MTHFR gene. No significant associations were noted between the DHFR 19-bp deletion genotype and breast milk concentrations of 5-methyl-THF and 5-formyl-THF.

## Discussion

To the best of our knowledge, this is the first study reporting folate forms and their association with common genetic variants affecting folate metabolism in a population where folate deficiency is a public health problem and where mandatory folate fortification does not take place. Our observations on high serum 5-formyl-THF at baseline, across pregnancy, and in cord blood and breast milk of women, as well as its rapid increase with a high dose of FA and its association with genetic variants following FA supplementation during pregnancy, are worth attention.

### Folate-Dependent One-Carbon Metabolism

**Figure 4:**
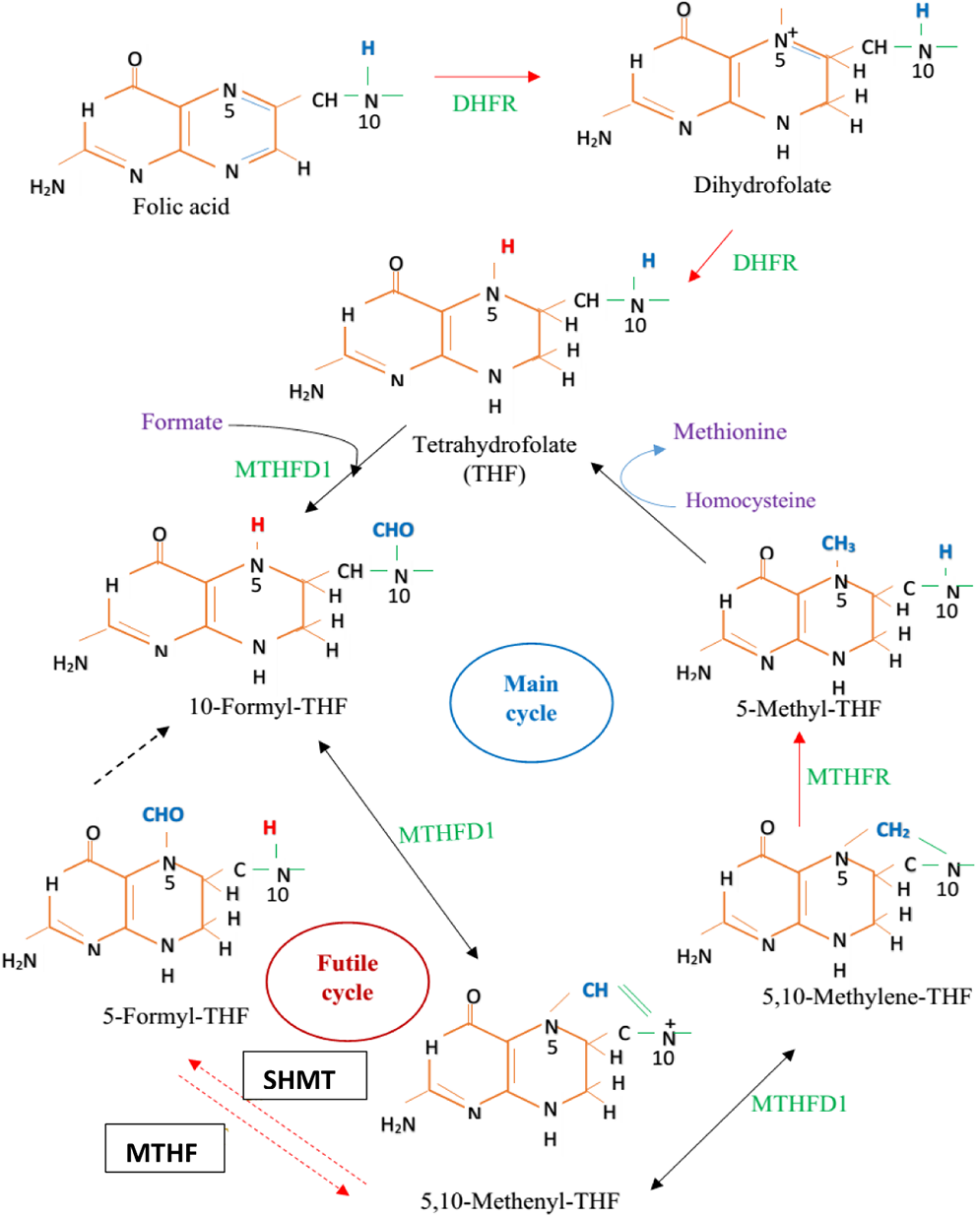
Folate-dependent one-carbon metabolism. Dihydrofolate reductase; MTHFD1, methylenetetrahydrofolate dehydrogenase 1; MTHFR, methylenetetrahydrofolate reductase; SHMT, serine hydroxymethyltransferase; MTHFS; methylenetetrahydrofolate synthase (the red line indicates the rate-limiting reactions). The enzyme DHFR converts folic acid to the active form of THF. THF is converted to 5,10-methylene-THF in the cytosol by a trifunctional enzyme, MTHFD1, following several steps of conversion. Finally, 5,10-methylene-THF is formed by the activity of methylene-THF dehydrogenase. All these pathways are bidirectional. MTHFR irreversibly reduces 5,10-methylene-THF to 5-methyl-THF. In addition to the main cycles, a futile cycle forms and catalyzes 5-formyl-THF using the enzymes serine hydroxymethyltransferase and 5,10-methenyl-THF synthetase, respectively.

Folate-dependent one-carbon metabolism is a tightly interconnected metabolic network (Figure 4). It is modulated by several folate coenzymes by accepting or donating one-carbon units.^21^ Tetrahydrofolate (THF) is the active form of folate, which is substituted with one of six different one-carbon units, such as serine, glycine, sarcosine, dimethylglycine, histidine, and formate. The accepted one-carbon unit exists in three different oxidation states. These oxidation states are equivalent to methanol, formaldehyde, and formate. 10-Formyl-THF, 5-formyl-THF, and 5,10-methenyl-THF carry one-carbon units at the oxidation state of formate. 5-Methyl-THF and 5,10-methylene-THF carry one-carbon units at the oxidation states of methanol and formaldehyde, respectively. These folate forms can be interconverted by cellular enzymes using nicotinamide adenine dinucleotide phosphate (NADP) or nicotinamide adenine dinucleotide (NAD) as cosubstrates (Zheng and Cantley, 2019). Formate and serine are the primary one-carbon sources for cytosolic and nuclear folate metabolism.^22^ In mitochondria, serine and glycine are converted to formate. This formate navigates to the cytosol and nucleus, where it is condensed with THF to form 5,10-methylene-THF in the cytosol. This reaction is catalyzed by a trifunctional enzyme, methylene-THF dehydrogenase 1 (MTHFD1). The MTHFD1 enzyme possesses formyl-THF synthetase, methenyl-THF cyclohydrolase, and methylene-THF dehydrogenase activities. First, the formyl-THF synthetase forms 10-formyl-THF. Subsequently, the methenyl-THF cyclohydrolase forms 5,10-methenyl-THF. Finally, 5,10-methylene-THF is formed by the activity of the methylene-THF dehydrogenase. All these pathways are bidirectional.^23^ In addition to this, 5,10-methylene-THF is also synthesized by vitamin B_6_-dependent serine hydroxymethyltransferase (SHMT). Methylene-THF reductase (MTHFR) is a rate-limiting enzyme that irreversibly reduces 5,10-methylene-THF to 5-methyl-THF, which is reported to be the predominant form of folate in blood circulation (Lan et al., 2018). 5-Methyl-THF donates its methyl group to homocysteine and is involved in the remethylation of homocysteine to methionine.

In addition to the main cycles, a futile cycle produces 5-formyl-THF. 5-Formyl-THF is not directly used as a cofactor in folate metabolism. However, it is the most stable reduced form of folate and is considered a storage form in mammalian cells. Serine hydroxymethyltransferase catalyzes the conversion of 5,10-methenyl-THF to 5-formyl-THF.^24^ 5,10-Methenyl-THF synthetase (MTHFS) is the only known mammalian enzyme that utilizes 5-formyl-THF. It converts 5-formyl-THF into 5,10-methenyl-THF in an irreversible ATP-dependent reaction.^25^

Synthetic FA cannot play a direct role in folate metabolism. It has to undergo a two-step enzymatic conversion to become active THF, which is involved in folate metabolism. The enzyme that converts FA to THF is dihydrofolate reductase (DHFR).^26^

### High Concentration of Baseline Serum 5-Formyl-THF

Our study found significantly higher serum 5-formyl-THF levels (41.6–220.7 nmol/L) compared to previous reports in pregnant women (0.23–3.39 nmol/L).^27,28^ It is important to note that the previous studies were conducted in Western populations with sufficient folate stores, where 5-formyl-THF accounted for only 1–2% of total serum folate, while 80–90% was 5-methyl-THF, the predominant form. Additionally, the RBC folate values in our study (371.6–906.4 nmol/L) were much lower than those reported in Canadian pregnant women (2266–4532 nmol/L) and other individuals, despite FA supplements or fortification.^29,30^ These differences highlight population-specific variations in folate levels, and associations between folate forms, age, race, and lifestyle have been noted in other studies.^9,31,32^

A model simulation suggests that, in folate deficiency, the futile cycling of 5-formyl-THF can be activated.^22^ Under normal conditions, 5-methyl-THF exerts feedback inhibition on the enzyme SHMT, which catalyzes the irreversible conversion of 5,10-methenyl-THF to 5-formyl-THF. However, in a low folate state, where 5-methyl-THF levels are reduced, this feedback inhibition is lifted, potentially increasing the synthesis of 5-formyl-THF. Hence, the observed unusually high levels of 5-formyl-THF in our study participants at baseline, who were not on any folate supplements, probably owing to low folate status. Moreover, variants of MTHFS or SHMT and the irregular pool of NADPH might also contribute to the high concentration of 5-formyl-THF observed in our population.^25,33^

### Rapid Increase in 5-Formyl-THF Following FA Supplementation

Following FA supplementation, 90% of women with folate deficiency replenished their folate stores (RBC folate > 342.2 nmol/L) and continuously increased their RBC folate throughout pregnancy, despite the difference in the supplemented dose, which is not a new observation.^34^ However, we also noted a rapid increase in 5-formyl-THF, the unusable form, when supplemented with FA. This observation is worrisome, as it also raises questions whether the increase in RBC folate is due to an increase in usable folate forms.

**Figure 5:**
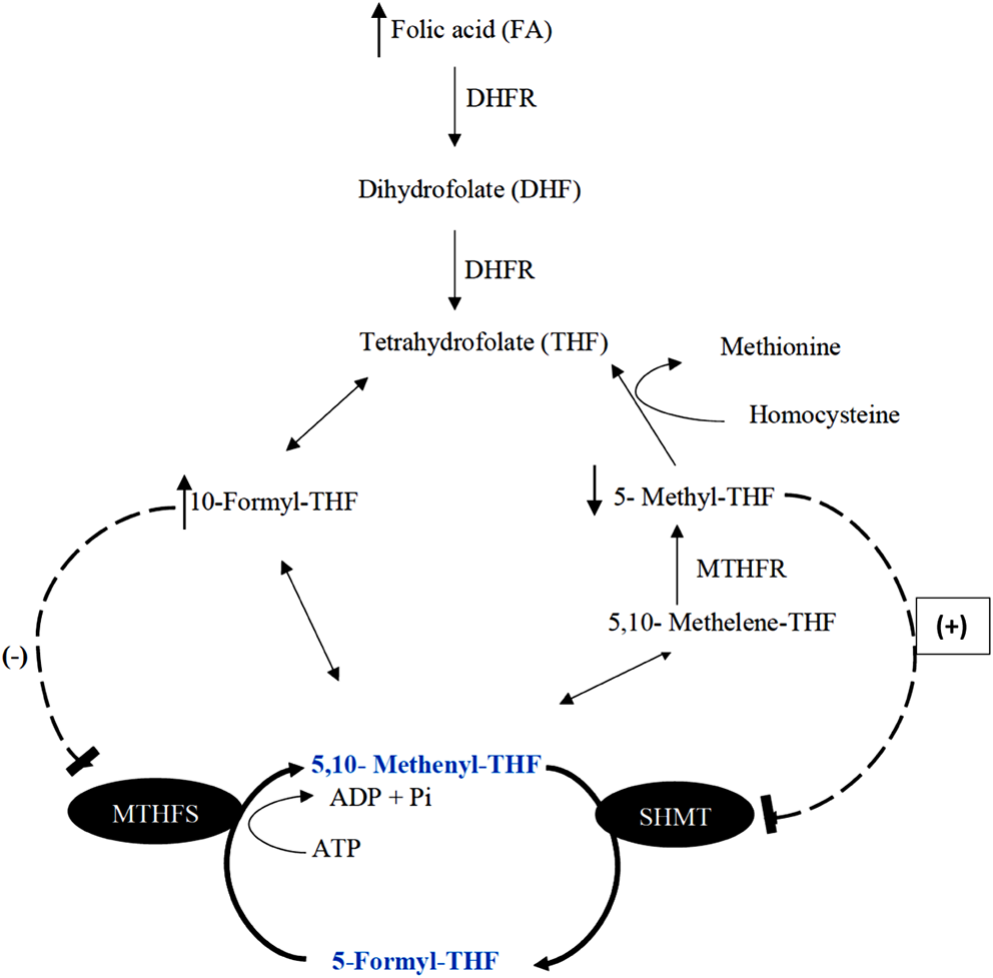
Pathway illustrating the formation of elevated 5-formyl-THF. DHFR: dihydrofolate reductase, MTHFS: methenyltetrahydrofolate synthetase, SHMT: serine hydroxymethyltransferase, and MTHFR: methylenetetrahydrofolate reductase. The dotted lines indicate feedback inhibition and activation. High intake of FA accelerates the production of 10-formyl-THF, which acts as an inhibitor of MTHFS—the enzyme that converts 5-formyl-THF into 5,10-methenyl-THF. As a result, 5-formyl-THF begins to accumulate. Meanwhile, the reduced formation of 5,10-methenyl-THF leads to decreased production of 5-methyl-THF, a known inhibitor of SHMT. The decline in 5-methyl-THF elevates the feedback inhibition on SHMT, further contributing to the accumulation of 5-formyl-THF.

High intake of FA leads to an increase in the folate metabolite 10-formyl-THF (Figure 5). This metabolite can inhibit the enzyme methenyltetrahydrofolate synthetase (MTHFS), which normally converts 5-formyl-THF back into 5,10-methenyl-THF. When MTHFS is inhibited due to elevated levels of 10-formyl-THF, the conversion of 5-formyl-THF is blocked or slowed down, resulting in the accumulation of 5-formyl-THF.^35^ This explains our observation of a rapid increase of 5-formyl-THF levels in our study participants following FA supplementation. Interestingly, we noted that the increase in 5-formyl-THF was dose-related, as the 5-formyl-THF levels subsequently reduced when lowering the FA dose from 12 weeks of gestation. Similar to our observations, early evidence showed a marked increase in urinary excretion of 5-formyl-THF in humans and rats fed with high synthetic FA, and the conversion of FA to 5-formyl-THF occurred in the rat liver.^36^

### Undetectable 5-Methyl-THF and FA in Serum

5-methyl-THF and FA in serum samples were below the level of quantification in our women. Although the limit of detection (LOD) we achieved (0.6 nmol/L) is higher than the LOD achieved by the CDC (0.06 nmol/L), it can vary based on interlaboratory variations and sample processing.^37^ However, a high LOD is unlikely to affect the detection of 5-methyl-THF even in folate-deficient individuals. A LOD of 1.5 nmol/L (0.66 ng/mL) has been reported for the detection of 5-methyl-THF in human serum samples.^38^ Further, participants in the present study ingested their FA supplements at night between 20:00 and 22:00 h, and venous blood samples were collected 12–14 h postsupplementation the following morning. Pharmacokinetic studies have demonstrated that plasma concentrations of 5-methyl-THF and unmetabolized FA reach their peak within 1–3 h following oral intake and subsequently decline due to distribution, metabolism, and excretion. The delayed timing of blood sample collection in this study may have coincided with the postabsorptive phase, potentially contributing to the absence of detectable circulating 5-methyl-THF. Furthermore, it has been reported that FA is reduced and methylated to form 5-methyl-THF within approximately 85 min, after which it is rapidly distributed to peripheral tissues, including the placenta, mammary glands, and other metabolically active sites. These factors collectively may account for the nondetection of 5-methyl-THF in the sampled circulation.^26^ In addition, the nonfasting status of participants at the time of blood collection may have influenced circulating 5-methyl-THF concentrations. Postprandial metabolic activity can alter folate absorption, hepatic metabolism, and tissue distribution, introducing variability into plasma folate levels. The presence or absence of dietary folate, as well as other dietary components that may interfere with folate metabolism, can further modulate the bioavailability and clearance of 5-methyl-THF.

Furthermore, we cannot rule out the possibility of oxidation of 5-methyl-THF, as it is much less chemically stable. This is further evidenced by the nondetection of 5-methyl-THF in folate-sufficient women in our study. Although we have taken all necessary precautions, from blood drawing to sample storage to minimize degradation of folate, such as careful handling of samples under dim light, along with immediately covering the tubes with aluminum foil and storing the samples in dark boxes at a nonacidic −80 °C temperature; the environmental conditions in a tropical country like Sri Lanka may have affected the stability of folate forms. Degradation during sample processing is unlikely, as the recovery and accuracy of 5-methyl-THF are >80%. The detectable concentration of 5-methyl-THF in breast milk confirms the accuracy of the analytical method. Conversely, a recent report has shown higher in vivo oxidation of 5-methyl-THF (MeFox), linking it to obesity, inflammation, and genetic predisposition.^39^ No studies have evaluated the changes in MeFox during pregnancy. These findings might indicate a possible increase in MeFox in vivo in our women, who have been identified with low-grade inflammation, possibly due to being overweight.^15^ These claims may be confirmed by the analysis of MeFox and the use of a standard reference material. However, limited resources constrained the analysis. The rapid elimination of UMFA^40^ might be the reason for the immeasurable FA in the circulation of our women.

### Association of Genetic Polymorphism and 5-Formyl-THF

The MTHFR C677T polymorphism, especially the presence of the T allele (as seen in CT and TT genotypes), is associated with reduced MTHFR enzyme activity. This can lead to decreased production of 5-methyl-THF, impairing folate metabolism and homocysteine remethylation. The association noted between the MTHFR C677T polymorphism and serum 5-formyl-THF follows the computational model presented by Misselbeck et al.^41^ The MTHFR C677T polymorphism leads to reduced activity of the MTHFR enzyme and therefore decreases the formation of 5-methyl-THF. Thus, it removes the feedback inhibition on SHMT and increases the folate flux toward the futile cycle (Figure 5). Inconsistent with this, another study noted higher levels of formylated THF in RBCs of individuals with the MTHFR polymorphism.^10^ Besides, the observations in circulatory folate metabolites and the associations observed with the MTHFR677CT/TT genotype, including higher 5-formyl and lower 5-methyl levels in breast milk, also align with the simulations by Misselbeck et al.

### Effect of 5-Formyl-THF Accumulation

The accumulation of 5-formyl-THF may have a significant physiological role in altering glycine, purine, and thymidylate synthesis, as it is an inhibitor of SHMT and also affects homocysteine remethylation.^42,43^ Hence, the regulation of the 5-formyl-THF futile cycle is critical to maintaining one-carbon homeostasis, especially during pregnancy, when rapid proliferative stages in development take place.^43^ Since folate deficiency is still a public health problem in Sri Lanka and other Asian countries, many nations, including India, have started FA fortification, and Sri Lanka is planning to launch it. However, the recommendations on safe intakes and reference ranges of FA established in these countries are based on data from Western populations. The studies showing the impact of FA fortification are limited in these countries and mainly focus on anemia. Our findings on high 5-formyl-THF highlight the importance of population dynamics. Hence, we recommend considering folate deficiency and genetic variations in key folate enzymes while establishing recommendations on safe intakes and reference ranges of FA. Further, the measures of the impact of FA supplementation should include all forms of folate, as the increased RBC folate in our study women appears to be due to the increase of futile forms in RBCs. Moreover, the dose-dependent effect in our study emphasizes the importance of using the lowest possible dose in supplementation/fortification programs, which may also have clinical implications. In the given background, populations may not be receiving the full potentiated benefits from widely used FA supplements.

### Strength and Weakness of the Study

The strength of our study is that we assessed the impact of FA supplementation on different folate forms in a cohort of pregnant women in a population where folate deficiency is a public health problem. Since the prevalence of NTD is 13–20 per 10 000 live births and anemia in our population is 34%, withholding supplementation from any woman during pregnancy would be unethical.^44,45^ We measured folate forms in subsamples due to limited resources, which limited the interpretation of our study. Although small (*n* = 50), the sample size fulfilled the requirements of 80% power to detect a 20% clinical reduction in folate deficiency following supplementation. The sample required for analyzing folate forms was 48 when the posthoc test was applied to the baseline data. However, it would have been appropriate to conduct the study in a larger population using fasting biological samples and analyzing other folate metabolites, and examining the catabolite would increase the understanding of folate metabolism and the distribution of folate forms in pregnant women.

## Conclusion

Our findings on high 5-formyl-THF highlight the importance of population dynamics, and the associations observed between folate forms, FA dose, and genetic variants in folate metabolism emphasize the caution to be taken while using high doses of FA in clinical practice and in establishing recommendations on safe intakes and reference ranges.

## Supporting Information Available

Analysis of folate forms via liquid chromatography–mass spectrometry (LC-MS/MS); stock solution preparation; sample preparation; calibration of spiked serum and breast milk standards and quality control samples; chromatography conditions; precision and recovery; precision and accuracy of serum folate form assay (Table S1); precision of breast milk folate form assay (Table S2) (PDF)
