# Supplementary material for: 5-Formyltetrahydrofolate in a Cohort of Pregnant Women Following Folic Acid Supplementation
Source: ACS Omega. 2025 Apr 28;10(18):18733–43. doi: 10.1021/acsomega.5c00251 (PMC12079205; doi:10.1021/acsomega.5c00251)
Supplement: Supplementary file 1 — ao5c00251_si_001.pdf [file ao5c00251_si_001.pdf]

Supplementary file

5-formyltetrahydrofolate in a cohort of pregnant women following folic acid supplementation

Author name

Miruna Sudharshani Kalaimani Rabindrakumar [a1], Veranja Karunaratne [b], Carukshi Arambepola [c], Vijay Pal Singh [d], Sharmila Jayasena [a], V. Pujitha Wickramasinghe [e], Tharanga Thoradeniya[a*]

Author affiliation

[a] Department of Biochemistry and Molecular Biology, Faculty of Medicine, University of Colombo, 25 Kynsey Rd, Colombo 00800, Sri Lanka
[b] Department of Chemistry, Faculty of Science, University of Peradeniya, University of Old Galaha Rd, Peradeniya 20400, Sri Lanka
[c] Department of Community Medicine, Faculty of Medicine, University of Colombo, 25 Kynsey Rd, Colombo 00800, Sri Lanka
[d] CSIR-Institute of Genomics & Integrative Biology, Academy of Scientific and Innovative Research, New Delhi, Delhi 110025, India
[e] Department of Paediatrics, Faculty of Medicine, University of Colombo, 25 Kynsey Rd, Colombo 00800, Sri Lanka

[*] tharanga@bmb.cmb.ac.lk

[1] Current Affiliation – Department of Life Sciences, Faculty of Science, NSBM Green University, Homagama, Sri Lanka. Email – miruna.r@nsbm.ac.lk

Analysis of folate forms via liquid chromatography-mass spectrometry (LC-MS/MS)

We measured the concentration of 5-methyl-THF, 5-formyl-THF and FA in serum and breast milk based on the method developed by Doly et al 2017 using LC-MS/ MS. The quantification of the mentioned folate forms was done by the SCIEX QTRAP – 5500 LC-MS/MS available at Dabur Research Foundation, Ghaziabad, Uttar Pradesh, India (GLP certified laboratory) [18,19].

Stock solutions preparation

The stock solution of 5-methyl-THF (1mg/mL) and 5-formyl-THF (1mg/mL) were prepared in 100% methanol. The stock solution of FA (1mg/L) was prepared in Milli-Q water containing 10% ammonium solution. The stock standard solutions were diluted in diluent containing 1:1 50mM ammonium acetate and methanol with 10mg/mL mercaptoethanol to prepare the series of working standard solutions. The concentration of working standard solutions was ranged from 0.07 to 1.0 µg/L. Aliquots (2.5 µL) of working standard solution were further diluted in blank charcoal treated serum/ breast milk (47.5 µL) to obtain calibration spiked serum/ breast milk standards ~0.003 – 0.5µg/mL. The stock solution of internal standard was prepared in Milli-Q water containing 10% ammonium solution and diluted with 10% Orthophosphoric acid in acetonitrile to prepare the 0.5µg/mL of the working solution.

Sample Preparation

The folate forms in serum and breastmilk samples were extracted via a protein precipitation method using 10% orthophosphoric acid in acetonitrile. To an aliquot of 47.5µL serum/calibrator/ quality controls, 2.5 µL internal standard working solution was added. After vortex for 5 minutes, centrifuged at 13000rpm for 5minutes. The supernatant was transferred to the clean tube and

100μL ammonium acetate buffer containing 1mg/mL of mercaptoethanol was added. The sample was vortex and transferred into auto sampler vials for injection into LC-MS/ system. Similar sample preparation was carried out for breast milk samples, except the blank preparation. Packaged branded milk sample was used as a blank for the calibration curve and QC preparation. The preparation of calibrators, quality controls and samples were carried out under dim light. All the Eppendorf tubes were wrapped in an aluminium foil.

Calibration spiked serum and breast milk standards and quality control samples

Calibrator and control samples were processed together with serum samples as described in the sample preparation. The calibrators were included in each batch of samples at concentrations of 0, 1.4, 2.7, 5.5, 11.4, 22.7, 45.5, 90.9 and 193.2 nmol/L for 5- methyl THF and FA and 0, 2.7, 5.5, 11.4, 22.7, 45.5,90.9, 181.8, and 386.4 nmol/L for 5-formyl-THF. The method was validated by running precision and accuracy batches of internal quality controls. Quality control samples were prepared at four different concentrations (1.4, 5.7, 45.5 and 187.5 nmol/L for 5-methyl-THF and FA and 1.4, 2.7, 45.5 and 187.5 nmol/L for 5-formyl-THF in both serum and breast milk. In addition, to each batch of samples, controls at four different concentrations from each folate forms were analyzed.

Chromatography conditions

LC-MS/MS analysis was carried out using the SCIEX QTRAP – 5500 LC-MS/MS system. The samples were chromatographed on a Zorbax C18 column (150 × 4.6 mm i.d., 5μm; Agilent). The mobile phase consisted of 0.5% acetic acid (solvent A) and 0.1% of formic acid in acetonitrile

(solvent B) according to the following program: 0-0.2 min (90% A), 3.5- 4.0 min (50% A) and 4.2 – 5.0 min (90% A). The total run time was 5 minutes with an injection volume of 25µL and the flow rate was set at 0.6mL/min. The auto sampler temperature was 10°C.

A triple–quadrupole mass spectrometer with trap function (API 5500 QTRAP, Applied BioSystems, SCIEX Toronto, Canada) and a turbo iron spray source was used. Positive ion electrospray ionization at 550°C with nitrogen as nebulizing gas was used for ionization. The MRM mode of the selected ion at the first (Q1) and third (Q3) quadrupole were used for detection of the analytes. Direct infusion of a single standard solution in water into MS was performed to choose the fragmentation patterns [m/z (Q1) → m/z (Q3)] of each analyte. The MRM conditions were further optimized to obtain maximum sensitivity for the analyte of interest.

Precision and recovery

The intra-assay CV was assessed by the quantification of six replicates of QC samples at four concentration levels on the same day. Inter-assay CV was assessed by the quantification of duplicates of QC samples on three consecutive days in three analytical batches. Intra and inter-assay CVs of serum samples are shown in Table S1, and the CVs were below 10%. Similar assay procedures were followed to assess the intra and inter-assay CVs of breast milk folate forms (Table S2). Further, an in-house prepared serum and breast milk pool samples were used at each analytical run and the CVs for both serum and breast milk pool for all three analytes were <6%. Recovery was performed by spiking serum and breast milk samples with three different levels. Mean recovery for 5-methyl-THF, 5-formyl and FA were >80%.

Supplementary Table S1. Precision and accuracy for serum folate form assay

Compound	High control			Medium Control			Low Control			LOQ control		
	Mean (SD) nmol/L	CV (%)	Accuracy	Mean (SD) nmol/L	CV (%)	Accuracy	Mean (SD) nmol/L	CV (%)	Accuracy	Mean (SD) nmol/L	CV (%)	Accuracy
Precision within day												
5- Methyl-THF	187.5 (12.5)	6	98.3	45.7 (2.7)	5.9	95.2	5.7 (0.2)	4	93.1	1.5 (0.07)	4.6	91.2
5- Formyl-THF	193.6 (10.2)	5.2	97.5	45.9 (3.2)	6.9	93.1	2.7 (0.9)	3.3	96.2	1.3 (0.09)	6.8	95.6
Folic acid	187.0 (3.3)	7.5	98.1	45.7 (1.6)	3.4	95.2	5.5 (0.2)	3.8	98.2	1.4 (0.07)	4.8	97.3
Precision day to day												
5- Methyl-THF	82.4 (7.5)	9.10	100.3	43.0 (3.6)	8.4	91.2	5.7 (0.2)	4	91.1	1.4 (0.14)	10	90.2
5- Formyl-THF	85.5 (8.7)	10.1	97	47.5 (4.3)	9.0	90.1	2.8 (0.9)	3.2	93.4	1.1 (0.07)	5	85.6
Folic acid	82.4 (4.7)	5.7	100.1	45.7 (2.0)	4.5	98.1	5.9 (0.2)	3.8	102.3	1.1 (0.1)	10	84.5

Supplementary Table S2 Precision of breast milk folate form assay

Compound	High control			Medium Control			Low Control			LOQ control		
	Mean (SD) nmol/L	CV (%)	Accuracy	Mean (SD) nmol/L	CV (%)	Accuracy	Mean (SD) nmol/L	CV (%)	Accuracy	Mean (SD) ng/mL	CV (%)	Accuracy
Precision within day												
5-Methyl-THF	185 (12.2)	6.6	96.3	50.2 (2.7)	6.7	96	5.5 (0.1)	2.4	93.1	1.5 (0.09)	6.2	91.2
5-formyl-THF	193.9(11.1)	5.2	94.3	45.5 (3.0)	6.5	94.1	2.7 (0.5)	3.3	96.2	1.4 (0.04)	3.2	95.6
Folic acid	185.2 (8.0)	4.3	96.1	45.7 (0.9)	1.9	93.2	5.2 (0.04)	2.2	98.2	1.4 (0.09)	6.3	97.3
Precision day to day												
5- Methyl THF	187.0 (14.8)	7.8	97.3	43.4 (2.5)	5.7	92.2	5.7 (0.2)	4	90.1	1.4 (0.09)	6.5	92.2
5- Formyl THF	193.8 (12.5)	6.4	95.6	45.7 (3.0)	6.4	89.1	2.8 (0.9)	3.2	97.4	1.5 (0.09)	6.3	89.2
Folic acid	187.5 (9.8)	5.2	89.1	45.9 (1.4)	2.9	93.1	5.2 (0.1)	3.8	96.5	1.5 (0.07)	4.6	90.2
